# Adapting an Evidence-Based, Early Childhood Parenting Programme for Integration into Government Primary Health Care Services in Rural Bangladesh

**DOI:** 10.3389/fpubh.2020.608173

**Published:** 2021-01-18

**Authors:** Syeda Fardina Mehrin, Jena Derakshani Hamadani, Nur-E Salveen, Mohammed Imrul Hasan, Sheikh Jamal Hossain, Helen Baker-Henningham

**Affiliations:** ^1^International Centre for Diarrhoeal Disease Research, Bangladesh (icddr,b), Dhaka, Bangladesh; ^2^School of Psychology, Bangor University, Bangor, United Kingdom

**Keywords:** early childhood, low- and middle-income countries, parenting programme, adapting interventions, integrated services, Bangladesh, psychosocial stimulation

## Abstract

This paper describes the process of adapting an early childhood development programme, with proven effectiveness in Bangladesh, for integration into government health services in rural Bangladesh. Through a three-stage process, we adapted an evidence-based, home-visiting, programme (Reach-Up and Learn) for delivery in government health clinics by government health staff as part of their regular duties. Stage one involved preparing an initial draft of two parenting interventions for use with: (1) pairs of mother/child dyads, and (2) small groups of mother/child dyads. In stage two, we piloted the adapted interventions in nine clinics with a total of twenty-seven health staff and 357 mother/child dyads. We used data from mothers' attendance, feedback from participating mothers and health staff and observations of parenting sessions by the research team to revise the interventions. Stage three involved piloting the revised interventions in six clinics with eighteen health staff and 162 mother/child dyads. We gathered additional data on mothers' attendance and used observations by the research team to finalize the interventions. Through this three-stage process, adaptations were made to the intervention content, process of delivery, materials, and engagement strategies used. The largest challenges were related to incorporating the parenting programme into health staff's existing workload and promoting mothers' engagement in the programme. We also simplified the content and structure of the curriculum to make it easier for health staff to deliver and to ensure mothers understood the activities introduced. This iterative piloting was used prior to implementing and evaluating the interventions through an effectiveness trial.

## Introduction

Worldwide 43% (250 million) children under-5 years of age in low- and middle- income countries (LMIC) are at risk of not attaining their full developmental potential due to poverty, malnutrition, and lack of appropriate early stimulation (1). There is evidence from many efficacy trials that early parenting interventions are effective in preventing the loss of children's development potential (2). The challenge now is to implement these interventions at scale to large numbers of disadvantaged children by integrating them into government health, nutrition, social security, or educational systems (3).

Poor Bangladeshi children show a significant cognitive deficit as early as 7 months of age compared to their more affluent peers, and the deficit grows larger as children reach 5 years of age (4). These deficits are likely to lead to long term effects on their educational attainment and adult productivity. There is thus an urgent need for national dissemination of effective parenting programmes to promote young children's development. The evidence-based, Jamaica home-visiting early childhood intervention programme (now called Reach-Up and Learn) was previously adapted for Bangladesh (5). The programme has been evaluated in three efficacy trials with undernourished children and has shown significant benefits to children's development and behavior and to mothers' parenting practices (6–9). The next steps are to extend the reach of this programme in Bangladesh, while maintaining its effectiveness.

The Bangladeshi government delivers primary health care through community clinics (CCs) in rural areas across the country. The CC is the lowest tier of the health facilities providing primary health care, family planning and nutritional services. There are over 13,000 CCs and they are established in all rural areas including remote and hard to reach areas. Each CC is staffed by three health workers: a Community Health Care Provider (CHCP), a Health Assistant (HA) and a Family Welfare Assistant (FWA). The CHCP works full-time in the clinic, and the HA and FWA spend 2–3 days at the clinic and the remaining days work in the community. CCs provide services for 6 days a week with operating hours from 9 a.m. to 3 p.m. The majority of CHCPs and HAs have Masters degrees and the FWAs have usually completed secondary (grade 10) level education. Most patients attend clinic in the morning and CC staff generally finish their regular schedule work around noon. Given the reach, structure and staffing levels of the community clinics, they provide a logical service for integrating parenting sessions within the routine work of the clinic.

The aim of this study was to adapt the Reach-Up and Learn home-visiting programme so that it could be implemented through the CCs with sessions conducted by government health staff as part of their routine duties. The beneficiary target group was mothers of undernourished children aged 6–36 months. We targeted undernourished children as undernutrition is an important risk factor for poor development, is relatively easy to measure, and has high prevalence in Bangladesh (10, 11).

## Methods

**Figure 1:**
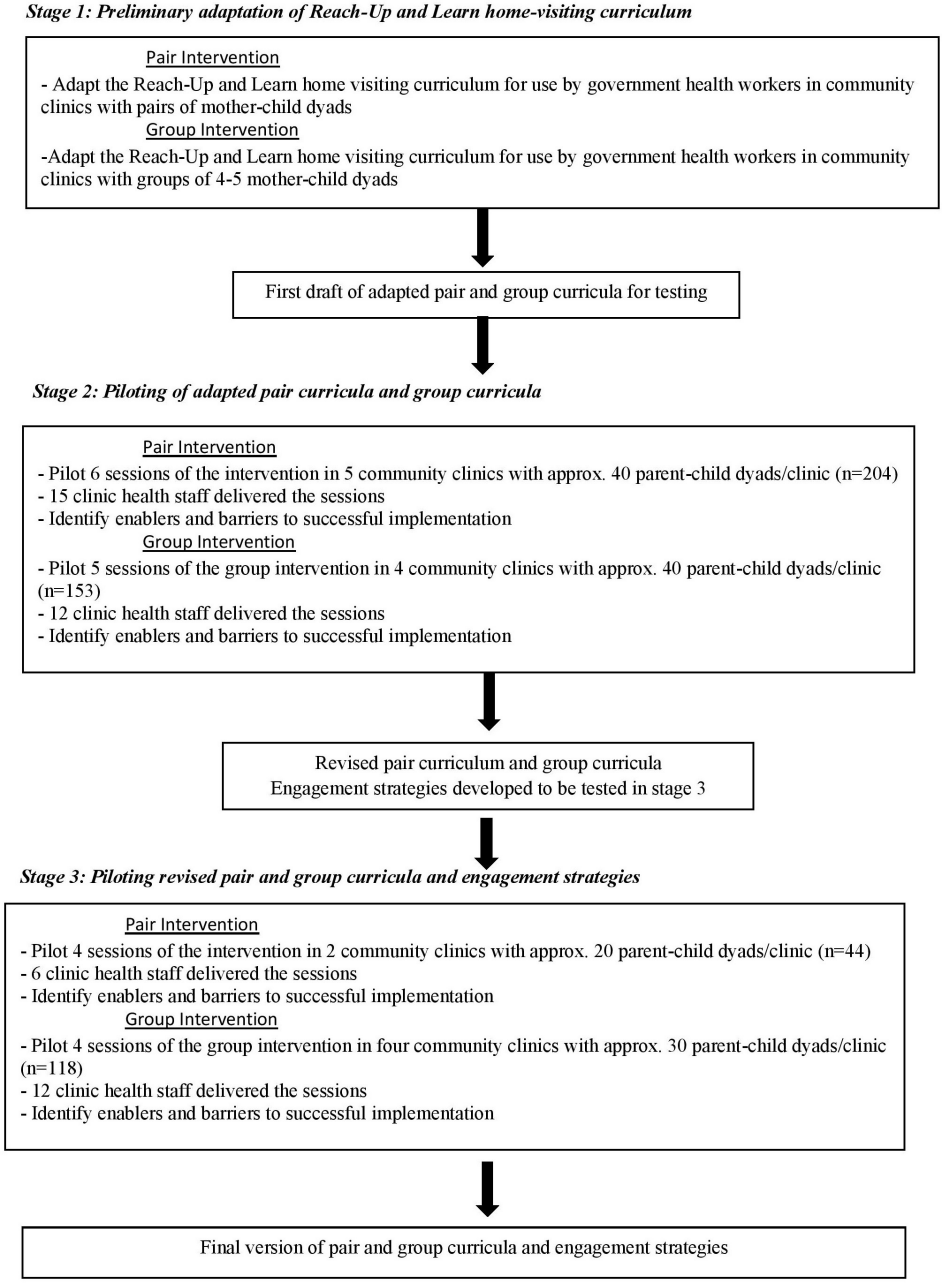
Stages followed in adapting Reach Up and Learn for integration into Government Health Services in Rural Bangladesh.

We developed two adapted versions of the Reach-Up and Learn home visiting programme for integration into the community clinic services including curricula for use with: (1) pairs of mother/child dyads, and (2) groups of four to five mother/child dyads. Both curricula have potential to extend the reach of the intervention, which was previously delivered with one mother/child dyad. The intervention development approaches used included: (1) the “target population centred” approach that involves incorporating the views and actions of the participants and delivery agents, and (2) the “implementation-based” approach that involves maximizing the likelihood that the intervention will be used in the real world (12). The study was conducted from July to December 2014 and consisted of three stages of formative research (Figure 1). In stage one, we developed a first draft of the two new curricula. In stage two, we piloted these curricula in nine CCs and identified enablers and barriers of implementation related to the content, structure, materials, and process of implementation. The data gathered was used to revise the curricula and process of delivery. In stage three, we piloted the revised curricula and tested two different participant engagement strategies in six CCs. The results of this study were used to finalize the two parenting interventions for testing in an effectiveness trial. Ethical clearance for the study was obtained from Institutional Review Board of the International Center for Diarrhoeal Disease Research, Bangladesh (icddr,b). Informed consent was obtained from all health staff and parents participating in the study.

### Stage One: Preliminary Adaptation of Reach-Up Home-Visiting Curriculum

Reach-Up and Learn is a home-visiting, play-based intervention for mothers of children aged 6 months to 4 years that was developed from the Jamaican early stimulation programme (13). This programme has evidence for long term benefits to children's IQ, educational and economic achievement, mental health, and involvement in violence (14, 15). It has been replicated in several low- and middle-income countries (16), including Bangladesh (7–10). The programme uses a structured curriculum, suitable for use by paraprofessionals, with developmentally appropriate activities organized to support weekly or fortnightly home visits. Emphasis is placed on parent-child interaction, praise for both parent and child, and encouraging parents to play and talk with their child during everyday routines and in structured play activities. The curriculum uses home-made toys (e.g., toys made from recyclable materials and soft toys such as doll, bean bag, ball), wooden blocks, picture books, puzzles, and games. One or two play materials are left in the home after each home-visit and then swapped for different materials at the next visit. To adapt this intervention for integration into the Bangladesh government health system we revised the curricula to make it suitable for use in the community clinic setting (rather than through home visits) and for use with either pairs or small groups of mother-child dyads to extend the reach of the programme. The content and structure of the first draft of the pair and group curricula is shown in Table 1. Each curriculum consisted of 12 parenting sessions, lasting between 45 min and 1 h, designed to be delivered fortnightly and suitable for mothers of children aged 6–36 months. The most important adaptations involved (1) reducing the number of play materials used in the intervention due to cost, transportation, and storage problems associated with providing a wide variety of materials, (2) adapting the play activities to make them suitable for children of a wider age range, (3) ensuring the activities were suitable for use in a clinic rather than a home setting.

**Table 1:** Development of the pair and group curricula.

	**Stage 1: First draft of adapted intervention**	**Summary of problems identified in piloting the adapted interventions in stage 2**	**Revisions made after piloting the interventions in stage 2**
Content and structure of pair curriculum	-Each session included four toy activities, one picture book activity, one language activity, a song and a developmental and nutritional message. - Developmentally appropriate toy, book and language activities were developed to be suitable for each month of a child's age (e.g., separate activities for 12 month olds, 13 month olds etc). - Nutritional and developmental messages and the song were conducted with both mothers together. - The curriculum was provided in one large manual divided into separate sections for each of the activities conducted during the session.	- It was not feasible to introduce four toy activities in each session because: (1) It was difficult for the health workers to manage so many different activities, (2) there was too many different activities for the mothers to remember and, (3) due to the limited space, it was not possible to have so many different toys in each session. - It was difficult for the health workers to use the large curriculum manual. - The language activities were largely based on discussion and the health workers would often miss or dedicate insufficient time to these activities. - There was limited interaction between the mothers, especially if the children were of different ages.	- We reduced the number of toy activities to two per session (rather than four). - We produced two separate curriculum manuals, one for the play activities and a different manual for the other activities to make it easier for the health workers to navigate. - We made the language activities more practical and concrete by for example, including objects, actions and games. - We included a greater focus on encouraging interaction between mothers. This included adding some common activities with the picture book, singing together and ensuring the nutritional and developmental message were delivered in a collaborative and interactive way.
Content and structure of group curriculum	- Each session included two toy activities, one picture book activity, one language activity, a song and a developmental and nutritional message. - Toy, book and language activities were developed for children in four age bands: 6–11 months, 12–18 months, 19–30 months, and 31–36 months. - Nutritional and developmental messages and the song were conducted with all mothers together. - The curriculum was provided in one large manual divided into separate sections.	- It was difficult for health workers to manage the children of different ages and to ensure that each mother-child dyad received the correct age-appropriate activity. - Mothers were confused about the specific activities that they were supposed to do with their child at the end of the session. - The nutritional and developmental messages were too long and the children would become tired and fussy. - The health workers would often miss or dedicate insufficient time to the language activities. - Health workers found it difficult to navigate the curriculum manual during the session.	- We reduced the number of toy activities to one per session (rather than two). - We asked mothers with children within the same age range (6–11 months, 12–18 months, 19–30 months, 31–36 months) to sit together so that it was easier for the health workers to conduct activities by child age. - We removed the developmental message from the curriculum. - We made the language activities more practical and concrete including objects, actions and games. - We prepared cards with a summary of each session to supplement the curriculum manual. These cards were used deliver the session.
Engagement of mothers	-Mothers of undernourished living within 45 min walk of the community clinic were recruited. - Health care staff set the schedule for the sessions.	- Poor attendance of mothers due to (1) the distance from the clinic, (2) attitude of family members to the sessions, (3) lack of interest in attending the sessions, (4) expectation of receiving incentives and/or food for attending. - Mothers were left waiting prior to the start of the session because (1) other mothers were late and (2) health staff were engaged with patients.	- We recruited mothers who lived within a 30-min walking distance from the clinic only. - We designed two different engagement strategies to be tested in stage three: (1) community motivational meetings, and (2) giving bottles of oil as an incentive. - Health staff and mothers meet to set a convenient time for the sessions when the clinic was likely to be relatively quiet.
Engagement of health workers	- In community clinics piloting the pair sessions, between 9 and 11 sessions/week were conducted. - In community clinics piloting the group sessions, 4–6 sessions/week were conducted.	- The additional workload for the health workers in the community clinics was demotivating and they reported that it was too burdensome to conduct so many sessions.	- We reduced the number mother/child dyads who would be enrolled in the programme (by 50% for the pair intervention and by 25% in the group intervention). - In community clinics conducting the pair intervention, the number of sessions was reduced to 4–5/week. - In community clinics conducting the group intervention, four sessions/week were conducted.

### Stage Two: Piloting of Adapted Pair and Group Curricula

#### Methods

The aim of stage two was to identify the enablers and barriers to implementing the adapted pair and group curricula within the community clinic network, using the existing health staff. This included factors relating to the content, structure, materials, and process of delivery of the curricula, the strategies used to recruit and engage the mothers in the session and the challenges faced by health staff in integrating the sessions into their regular workload.

##### Sample

The study was conducted in the Sonargaon sub-district, under Narayanganj district, located 80 km from Dhaka city. Sonargaon consists of 30 community clinics and nine clinics were purposively selected for easy accessibility and availability of transportation facilities. We screened all households with children aged between 6 and 33 months old living within 45 min walking distance from the selected clinics. All children were weighed and those with weight-for-age z-score of ≤-2SDs of the WHO growth standards were eligible to participate in the study. We enrolled up to 40 children in each clinic; when more than 40 children were identified, we randomly selected 40 children to participate. The programme was discussed with the mother and other members of the household who were present and the mother was invited to participate in the parenting sessions. Children with severe malnutrition (<-3SDs weight for age z-score) and children with disabilities were referred for specialist services and excluded from the study. Details of the number of clinics, health staff, and mother/child dyads participating in the piloting activities are shown in Figure 1.

##### Intervention Implementation

The research team provided 2 weeks training in how to conduct the parenting sessions to the Community Health Care Provider (CHCP), Family Welfare Assistant (FWA) and Health Assistant (HA) from each of the selected nine clinics. Training was conducted in two waves: health staff from five clinics were trained in the pair curriculum and health workers from four clinics were trained in the group curriculum. A member of the research team supervised the parenting sessions with a minimum of two visits per week to each clinic. During each visit, the supervisor assisted the health staff in preparing for the session, provided ongoing support during the session, and completed a monitoring form with input from the health staff to record enablers and barriers to programme implementation. During this pilot study, mothers attending clinics implementing the pair curriculum were offered a total of six parenting sessions, while five parenting sessions were offered to mothers attending clinics delivering the group curriculum. Each mother-child dyad attended fortnightly sessions. Health staff in pair clinics conducted 9–11 sessions/week and staff in group clinics delivered 4–6 sessions/week.

##### Data Collection

We collected data on parent attendance at each session and conducted semi-structured interviews with health care staff and a subsample of participating mothers. Twenty-three mothers were selected from two of the pair clinics based on session attendance to identify the enablers and barriers to attendance from the perspective of the mothers. The sample included five mothers who attended all six sessions, ten mothers who attended three sessions, four mothers who attended one session and four mothers who attended zero sessions. Nineteen mothers who had attended three or more sessions were selected from two of the group clinics to investigate the extent to which the mothers understood the activities that were introduced in the sessions. Mothers were asked to show the interviewer the toy, book, and language activity that had been introduced in the previous session and to describe the developmental and nutritional message. This data was used to assess if mothers remembered and understood the activities and messages from the session. Mothers were also asked how many times a week they conducted the activities at home. Mothers were interviewed at their home by a female member of the research team using a semi-structured questionnaire. Mothers' responses were written verbatim. Each interview lasted ~30 min. We also conducted two group discussions with clinic staff to explore the enablers and barriers to implementing the parenting sessions from their perspective. A semi-structured interview was used and covered enablers and barriers relating to the curriculum content, the materials used, the process of delivering the sessions, mother engagement, and integrating the sessions into the regular clinic activities. Nine health staff from pair clinics and eight health staff from the group clinics participated in these discussions. Two researchers conducted these interviews with one moderating the discussion and the second keeping detailed notes.

#### Results

Mothers' attendance declined over time in both pilot studies (Table 2). Table 3 shows the main reasons for non-attendance from the perspective of mothers in the pair clinics and the data on mothers' understanding of the activities from the interviews with mothers attending the group clinics. Many mothers expressed ambivalence about the sessions, describing the positive aspects of the sessions while also mentioning factors that limited their motivation to fully engage with the programme:

**Table 2:** Mothers' attendance at parenting sessions in stage 2 and stage 3.

**Stage 2 piloting: without specific engagement strategy**				**Stage 3 piloting: with specific engagement strategy**			
**Pair intervention** ***n*** **=** **204**		**Group intervention** ***n*** **=** **153**		**Pair intervention (motivational meetings)** ***n*** **=** **44**		**Group intervention (half liter bottle of oil)** ***n*** **=** **118**	
**Session**	***n* (%)**	**Session**	***n* (%)**	**Session**	***n* (%)**	**Session**	***n* (%)**
1	173 (85%)	1	121 (79%)	1	42 (95%)	1	100 (85%)
2	138 (67%)	2	100 (65%)	2	36 (81%)	2	108 (91%)
3	97 (47%)	3	82 (54%)	3	38 (87%)	3	109 (93%)
4	68 (33%)	4	83 (54%)	4	36 (81%)	4	109 (93%)
5	47 (23%)	5	67 (44%)				
6	30 (15%)						

**Table 3:** Supervisors', health staff and mothers' perceptions in stage two.

**Mothers' perceptions and understanding of the parenting sessions**			
**Interviews with mothers attending pair sessions**		**Interviews with mothers attending group sessions**	
**Mothers opinions on the session**	***n* = 23** ***n* (%)**	**Mothers understanding of the sessions**	***n* = 19** ***n* (%)**
Mother believes programme is important for her child's development	19 (82%)	Book activity reported by mother was appropriate to the child's age	10 (53%)
Mother reports other family members believe the programme is important	15 (65%)	Toy 1 activity reported by mother was appropriate to the child's age	12 (63%)
Likes the toys	10 (43%)	Toy 2 activity reported by mother was appropriate to the child's age	15 (79%)
**Mothers' reasons for missing sessions**	***n*** **=** **18** ***n*** **(%)**	Language activity reported by mother was correct for the child's age	12 (63%)
No incentive given	13 (72%)	Mother recalled the developmental message	12 (63%)
Family members discouraged attendance	7 (39%)	Mother followed the nutritional advice	15 (79%)
Sessions are not important for the development of their child	6 (33%)	Mother practiced the activity at home Every day 3–4 times a week 1–2 times a week	8 (42%) 10 (53%) 1 (5%)
Toys were not attractive and interesting	6 (33%)		
Live far from clinic	5 (28%)		
Involvement with earning sources	4 (22%)		
**Perceptions of health staff on the enablers and barriers to successful implementation of the parenting sessions**			
**Enablers** - CC staff believed that the activities introduced through the curriculum are suitable for young children and will help children's development. - Most CC staff enjoyed conducting the sessions and believed that conducting the parenting sessions was an appropriate part of their role.			
**Barriers** - Mothers expect to be provided with food supplements or medicine when they attend clinic. - Family members were not motivated well during the recruitment process and hence they were not always supportive of the mother attending the sessions. - Some mothers do not like the home-made toys and they find them unattractive. - Delivering so many toy activities is difficult. It is hard to keep the interest of the mothers and children and to organize the materials. - The curriculum manual was difficult to use during the session: it was too large and difficult to navigate between the sections. - It was difficult to conduct so many sessions and continue to meet the demands of their regular workload. - It was difficult to handle children of different age groups, especially in the group curriculum.			
**Observations of supervisors attending the sessions**			
**Enablers** -Health staff used many of the key training techniques while conducting the sessions including demonstrating the toy and book activities and the encouraging mothers to practice. - Health staff had a positive attitude to conducting the sessions and were willing to make time in their schedule to conduct the sessions.			
**Barriers** - Language activities were often given insufficient emphasis. The health staff tended to be more didactic in introducing these activities and rarely asked mothers to practice the activities with their child. - There was too much time spent on discussion, especially in the group sessions and the children became tired, fussy and restless when they were not engaged in activities. - The health staff found it difficult to organize the materials for the session in the small space available. - There were too many toy activities in the curricula and the health staff sometimes mixed up the toys and activities and delivered age-inappropriate messages. - The curriculum manual was unwieldly to use as the CC staff needed to switch between several sections This led to pauses in the session as the CC staff found the correct page and children and mothers became distracted and bored. - At times mothers and children would be required to wait for the CC staff as they continued to deal with patients attending the clinic. - In the group clinics, it was difficult to gather all mothers at the same time and this could lead to long waits for mothers and children who arrived first.

“*The session is very much and he enjoyed the sessions. But the clinic does not provide any vitamin syrup. If the clinic gives medicine, nutritious food and advice about nutrition and sickness then I will come to the sessions.” (Mother from pair clinic)*

In the group clinics, the majority of mothers (95%) reported doing the activities at home frequently with their child. However, the activities reported were not always age-appropriate, indicating that more clarity was needed during session delivery.

Key enablers and barriers from the perspective of the clinic staff and core observations from the supervisors are also given in Table 3. The clinic staff recognized the value of and gained professional satisfaction from conducting the sessions; their main concerns related to the increased workload.

“*I personally think the program is good. Sometimes I feel it is an extra work but not always. If children play well and do better then I feel good to see that the children are improving.” (Community Health Care Provider)*

Other barriers to intervention implementation were related to the complexity of the curricula. For example, there were too many activities included in each session, the curriculum manuals were hard to use, and health workers spent insufficient time on some activities. Health staff were also overly didactic during the parts of the session that involved discussion. Based on the data collected, we made revisions to both the pair and group curriculum to make the sessions easier to conduct and to encourage more participatory and interactive delivery methods, and we adapted the process of implementation to ensure the programme could be managed within the staff workload (see Table 1). In addition, due to the poor mother attendance, we limited eligibility for the programme to mothers who lived within a 30-min walk from the clinic and designed two recruitment strategies to be tested in stage three: (1) providing an incentive to attend the sessions, and (2) conducting community motivational meetings prior to restarting the parenting sessions.

### Stage 3: Piloting Revised Pair and Group Curricula and Engagement Strategies

#### Methods

The main aim of the stage three was to evaluate the effectiveness of two different engagement strategies on mothers' attendance. We also continued to monitor the quality of the parenting sessions to identify if the changes made to the curricula led to more successful implementation.

##### Sample

For financial and logistical reasons, the pilot studies in stage three were conducted in only six of the nine selected clinics. From the original sample of 40 parent-child dyads/clinic, we excluded parents who lived more than a 30-min walk from the clinic and then randomly selected 20 parent-child dyads from pair clinics and 30 parent-child dyads from group clinics to continue in the programme (Figure 1).

##### Engagement Strategies

In community clinics conducting the pair sessions, we organized community motivational meetings that were attended by village elites, local leaders, school teachers, health care staff and the selected mothers and their families. We provided psychoeducation around the importance of play and stimulation for child development and introduced the play materials used in the programme. The village leader also delivered a speech encouraging families to engage with the programme. In the community clinics conducting the group sessions, we offered mothers an incentive of half a liter of cooking oil for every session attended.

##### Intervention Implementation

A further four parenting sessions were offered to mother-child dyads in all six clinics participating in the stage three piloting activities. Mothers attended sessions once a fortnight and a total of 4–5 parenting sessions/week were conducted in pair clinics and 2–3 parenting sessions a week were conducted in group clinics. Supervisors continued to attend a minimum of two sessions per week in each community clinic to provide ongoing support to the health staff and to document the quality of the sessions.

##### Data Collection

Mothers' attendance at the sessions was recorded. Supervisors completed record forms on the enablers and barriers to implementation after each session.

#### Results

The adaptations to the curricula, including adaptations to the content, materials, and structure as shown in Table 1, led to higher quality implementation of the curriculum. Observations by supervisors indicated that the sessions were more organized and interactive and health staff were competent at conducting the age appropriate activities with each mother-child dyad. Sessions were usually held on time and the reduction in the number of sessions held per week made it easier for the health staff to schedule the sessions when the clinic was quiet.

Mothers' attendance was greatly improved with both engagement strategies used (Table 2). The incentive of a half liter bottle of oil was highly successful at motivating mothers to attend with average attendance above 90%. However, there were logistical problems in giving oil related to purchasing, delivery, storage, accountability, and record-keeping. Furthermore, other members of the community started to gather at the clinic to ask for oil and the health staff also expected to be given a bottle of oil after each session. The use of community motivational meetings led to a slightly lower, but still acceptable average attendance of above 80%.

### Final Versions of the Adapted Parenting Interventions

The final version of the curricula consisted of 12 fortnightly parenting sessions, suitable for children aged 6–36 months that were repeated every 6 months. Activities were divided into child age months for the pair curriculum and either 6- or 12-month age bands in the group curricula. Hence, when the sessions were repeated, most children would be introduced to activities in the higher age range. Each session included the following: (1) feedback from the previous session, (2) a song, (3) demonstration and practice of the toy, book and language activities, (4) developmental and/or nutritional message(s), and (5) review and reminder of home activities. Mothers were given at least one book and one toy during each session and these were swapped for a different book and toy at the next session. Given the cost and the logistical challenges associated with providing oil and the success of the community motivational meetings, we chose the latter strategy as the key engagement strategy to be used in the effectiveness trial. We also planned for quarterly refresher meetings within the community with small gifts provided to mothers with high attendance at the sessions to sustain mothers' engagement in the programme. We also decided to only recruit eligible mothers who lived within a 30-min walking distance from the community clinic. The revised curricula have since been evaluated with large benefits found for child cognition, language, motor development and behavior in addition to home stimulation, maternal knowledge of child rearing and maternal depression (17). Interestingly, the benefits were larger and more extensive than found in previous studies in Bangladesh possibly because the pair/group format provided social support and the health providers were better educated and more credible than previous home visitors.

## Discussion

In this brief research report, we describe the process of adapting an early childhood parenting intervention with proven effectiveness in the Bangladeshi context for integration into the government health service including delivery by existing health staff. Adaptations were required to take into account differences in the delivery location (clinic vs. home), delivery agents used (health care staff vs. persons hired by the research team), and the intended reach (higher number of mothers reached per delivery agent) of the intervention. The adaptation process involved two rounds of piloting and during the piloting process we (1) measured mothers' attendance at the sessions, (2) incorporated feedback from the intended beneficiaries (mothers) and the delivery agents (health staff), and (3) documented enablers and barriers to implementation through ongoing observations of the research team. Adaptations were made to the content of the curriculum, the materials used, the process of delivery, and the engagement strategies used. The paper illustrates the importance of thorough piloting of all aspects of an intervention, when moving from smaller-scale implementation in an efficacy trial to large scale implementation through an existing service. The value of investing resources to iteratively field test an adapted intervention in the context in which it is to be implemented is demonstrated by the large and wide-ranging benefits to children and mothers who later participated in an effectiveness trial (17).

There are examples in the literature relating to (1) transporting evidence-based parenting interventions across countries (18–20), (2) developing interventions specific for the context (21–23), and (3) integrating interventions into government services (19, 24). There is less literature on adapting an early childhood parenting intervention, with proven effectiveness in the setting, for wide-scale dissemination by integrating it into existing services. However, there are some common issues across this literature on developing, adapting and integrating early childhood interventions in LMIC.

Firstly, attention to staff workload is important, especially when integrating into an existing service. Through the piloting process, we aimed to incorporate the additional responsibilities into staff's existing duties without reducing their effectiveness in other aspects of their role, and without leading to staff demotivation. Other early childhood parenting interventions have reported that staff workload is problematic leading to high staff turnover (18) and/or a reduction in the number and duration of planned sessions (24). We designed a clinic-based intervention to facilitate the integration of the programme into the health staff's existing duties. Through piloting, we determined the number of weekly sessions each health worker could feasibly conduct.

Secondly, it is important to develop a feasible and effective strategy for engaging participants in the intervention. We found the strategies that we had previously used to engage mothers in a home-visiting intervention, were less effective at engaging mothers in a clinic-based intervention. The lack of an incentive and family members discouraging attendance were the most frequent barriers to mothers' engagement. These barriers have also been reported in a recent study in Bangladesh (20). We tried two specific engagement strategies, giving cooking oil as an incentive for attendance and community motivational meetings to engage all family members. Both strategies were effective although giving oil proved difficult due to logistical problems. The importance of community sensitization to increase participant engagement in parenting programmes has been reported in other studies (19, 20).

Thirdly, it is important to ensure sessions are simple for staff to deliver and the messages and activities are easy for mothers to understand. We found the curricula manuals were too complex for the health staff to use during the sessions, particularly for the group sessions. Navigating through the different sections of the manual took their attention away from the mothers and children, leading to lower participation and interest. We therefore designed a laminated summary card for use during the session. Also, when many activities were introduced during the session, mothers were unable to recall the content. A simple, streamlined intervention with user-friendly manuals is recommended to maintain the interest and engagement of participants and to maximize the likelihood that the intervention will be delivered with fidelity (20). Reducing the complexity of the intervention to make it easier to deliver is also likely to lead to higher motivation among the health staff (25).

Fourthly, using active learning and evidence-based behavior change techniques (e.g., demonstration, practice, positive feedback, social support) are key characteristics of effective early childhood programmes (26, 27). Through the piloting process, we identified opportunities for improving the intervention content, materials, and process of delivery to ensure that these techniques were embedded into the intervention design. For example, we adapted the content to promote mothers' interaction with each other and to ensure the sessions were collaborative and participatory. We also revised the activities that involved group discussion to make them more practical and concrete by incorporating games, objects, and actions to maintain participants' engagement. Using hands-on, practical activities also ensured that the health staff dedicated sufficient time to these activities.

Fifthly, it is important to decide on the target population for the intervention as it may not be feasible to offer a parenting intervention for all families in need. For example, in Malawi, health staff were only able to reach 14.2% of eligible children, with an early childhood development intervention (19). We targeted undernourished children but even so, we were limited by the capacity of the health clinics and the programme was not suitable for mothers living far from the clinic. Alternative strategies are required to reach all young disadvantaged children and their families.

Finally, incorporating participant feedback including feedback from potential beneficiaries and from the delivery agents is an important part of the adaptation process (20–23), in addition to using observations from the research team during intervention implementation (23). To adapt interventions for a new context, we recommend the use of iterative rounds of piloting with ongoing improvement and refinements to the intervention to promote the acceptability, relevance and feasibility of the intervention for participants and implementers.

## Data Availability Statement

The datasets generated in this article are not readily available because most of the data is not suitable for sharing as it is not fully anonymised. Requests to access the datasets should be directed to fardina.mehrin@iccdrb.org.

## Ethics Statement

The studies involving human participants were reviewed and approved by Institutional Review Board of the International Center for Diarrhoeal Disease Research, Bangladesh (iccdr,b). Informed consent was provided by all health staff and mothers participating in the study.

## Author Contributions

JDH and HB-H contributed to the conceptualization of the study and funding acquisition. SM, N-ES, JDH, MH, and SH contributed to project administration. SM, JDH, HB-H, N-ES, MH, and SH contributed to investigation. SM, HB-H, and JDH were responsible for data curation and data analysis. SM and HB-H were responsible for writing the original draft. All authors reviewed and edited the manuscript.

## Conflict of Interest

The authors declare that the research was conducted in the absence of any commercial or financial relationships that could be construed as a potential conflict of interest.
